# Acne in adulthood: A cross-sectional study of contributing hormonal, dietary, and environmental factors in adults in Puerto Rico

**DOI:** 10.1016/j.jdin.2025.11.006

**Published:** 2025-11-15

**Authors:** Itzamar Pastrana Echevarria, Alicia Báez Cruz, Alejandra Figueroa Moreda, Fabian Ramírez Rivera, Lymarie Aguila Gonzalez

**Affiliations:** a St. Luke’s Hospital Department of Graduate Medical Education Research Fellow, Ponce, Puerto Rico; b Medical Student, Universidad Central del Caribe School of Medicine, Bayamón, Puerto Rico; c Family Medicine Department, University of Puerto Rico, Medical Sciences Campus Dr. José Celso Barbosa, San Juan, Puerto Rico; d Department of Graduate Medical Education, St. Luke’s Hospital, Research Fellow Administrator, Ponce, Puerto Rico; e Dermatologist, Torre Medica I, Manatí, Puerto Rico

*To the Editor:* Acne Vulgaris ranks eighth in disease prevalence, affecting 9.4% of the population.^1^ Although commonly triggered during adolescence, it can occur at any age.^2^ In the United States, adult acne incidence is rising, affecting up to 15% of women.^2^ While adolescent males are more commonly affected, this trend reverses in adulthood, with 26% of women and 12% of men reporting acne in their 40s.^3^

Various factors have been implicated in adult acne, including hormonal, dietary, hygiene-related, stress, sleep and environmental influences. Although no definitive cause has been established, this study assessed their association in the Puerto Rican population, where unique behavioral and contextual factors may differ from the mainland United States.

An anonymous 22-item REDCap survey was distributed via social media and in a dermatology clinic in Manatí, Puerto Rico through promotional flyers with a QR code between April and July 2025. This IRB approved cross-sectional study used a convenience sampling method. A total of 328 surveys were initiated and 298 were completed, yielding a response rate of 90.9%. After applying inclusion criteria, 246 participants were included in the final analysis. Adult acne was defined as onset at ≥ 25 years of age.

As participation was voluntary and the survey title referenced acne, individuals with acne may have been more inclined to respond, introducing potential selection and response bias. Descriptive statistics were used to summarize demographic and clinical variables. Associations between categorical variables were evaluated using chi-square test, with significance set at *P* < .05. Analyses were performed using IBM SPSS Statistics.

Of the 246 participants: 172 with self-reported adult acne and 126 without. All participants self-identified as Hispanic. The overall sample was predominantly female (72%); however, the proportion of males who reported acne (60.9%) was higher than that of females (52.5%), diverging from U.S. trends (Fig 1). Most cases occurred between ages 25-34 years. Persistent (21.5%) and recurrent (22%) acne subtypes were most common. Notably, 74% of participants had never consulted a dermatologist, suggesting underrecognition or access barriers to care.

**Fig 1:**
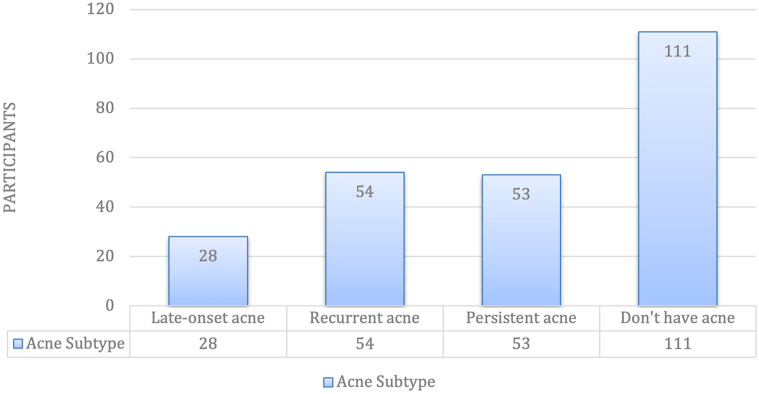
Prevalence of adult acne subtypes (*n* = 246). Recurrent (*n* = 54) and persistent (*n* = 53) were the most common subtypes, followed by late-onset acne (*n* = 28). A total of 111 participants reported not having acne.

Poor sleep quality was significantly associated with adult acne (*P* = .019), aligning with current literature.^4^ Inconsistent makeup removal also correlated with acne (*P* = .04), reinforcing clinical recommendations for proper skincare although no specific studies have evaluated a direct association between adult acne and not removing makeup at the end of the day.^5^ Use of non-comedogenic products showed a borderline association (*P* = .051), possibly reflecting reverse causality, though it remains advisable to avoid oil-based, pore-clogging products. Additionally, refined carbohydrates and dairy intake showed positive but non-significant trends, supporting hypotheses on diet-acne links. Moreover, environmental factors may contribute with nearly 30% reported flares during summer, aligning with data from tropical regions.

Future research using dermatologist confirmed diagnoses and prospective data could improve accuracy. This study offers novel insights into adult acne in Puerto Rico, emphasizing male prevalence, hormonal patterns, and lifestyle factors. These findings support culturally tailored approaches to acne care (Table I).

**Table I:** Association between stress level, sleep quality, and adult acne presence (*n* = 246)

Variables (*n*)	Acne development		*P*-value
	Yes (*n* = 135)	No (*n* = 111)	
Non-comedogenic skin care			
Didn’t know the existence (55)	25 (45.5%)	30 (54.5%)	.051
Don’t’ pay attention (61)	28 (45.9%)	33 (54.0%)	
Only when I see breakouts (24)	16 (66.7%)	8 (33.3%)	
Always use (106)	66 (62.3%)	40 (37.7%)	
Removal of makeup before sleep			
Doesn’t apply (55)	21 (38.2%)	34 (61.8%)	.04
Don’t use makeup (23)	13 (56.5%)	10 (43.5%)	
Sometimes (18)	13 (72.2%)	5 (27.8%)	
Generally, no (1)	1 (100%)	0 (0%)	
Yes, always (149)	87 (58.4%)	62 (41.6%)	
Bed pillow cover changing			
Don’t change them (12)	6 (50%)	6 (50%)	.912
2 times a month (27)	16 (59.3%)	11 (40.7%)	
Once a month (60)	33 (55%)	27 (45%)	
Once a week (121)	64 (52.9%)	57 (47.1%)	
Several times a week (26)	16 (61.5%)	10 (38.5%)	
Frequency of face-towel change			
Don’t change them (12)	6 (50%)	6 (50%)	.982
Once a month (24)	13 9 (54.2%)	11 (45.8%)	
Once a week (137)	75 (54.7%)	62 (45.5%)	
Several times a week (73)	41 (56.2%)	32 (43.8%)	
Using the same towel for body & face			
Use the same towel (141)	38 (57.6%)	28 (42.4%)	.822
Use them separately (66)	75 (53.2%)	66 (46.8%)	
Try to use them separately (39)	22 (56.4%)	17 (43.6%)	
Cleaning cellphone screen			
Rarely (48)	30 (62.5%)	18 (37.5%)	.312
When visibly dirty (87)	51 (58.6%)	36 (41.4%)	
Once a day (60)	28 (46.7%)	32 (53.3%)	
Multiple times a week (51)	26 (51%)	25 (49%)	
Frequency of facial washing a day			
Don’t do it often (10)	2 (20%)	8 (80%)	.074
When I need it (41)	18 (43.9%)	23 (56.1%)	
More than 2 times (22)	14 (63.6%)	8 (36.4%)	
Morning and when going to bed (115)	68 (59.1%)	47 (40.9%)	
Only in morning or night (58)	33 (56.9%)	25 (45.1%)	
Stress level			
Low (15)	8 (53.3%)	7 (46.7%)	.071
Moderate (137)	67 (48.9%)	70 (51.1%)	
High (94)	60 (63.8%)	34 (36.2%)	
Sleep quality			
Bad (51)	36 (70.6%)	15 (29.4%)	.019
Moderate (130)	70 (53.8%)	60 (46.2%)	

Chi-square analysis examining the relationship between perceived stress levels and sleep quality with adult acne presence. Values are shown as number of participants with acne (%). *P* = values < .05 were considered statistically significant.

## Conflicts of interest

None disclosed.

## References

[1] Tan J.K., Bhate K. (2015). A global perspective on the epidemiology of acne. Br J Dermatol.

[2] American Academy of Dermatology (2024). Skin conditions by the numbers [Internet]. Rosemont (IL): American Academy of Dermatology. https://www.aad.org/media/stats-numbers.

[3] Zaenglein A.L. (2018). Acne vulgaris. N Engl J Med.

[4] Putri W.E., Hilda N.F., Kusumah F.R. (2024). The relationship between fast food, sleep patterns, and facial hygiene with the severity of Acne vulgaris. Biomol Health Sci J.

[5] (2023). American Academy of Dermatology. I have acne! is it okay to wear makeup? [Internet]. Rosemont (IL): American Academy of Dermatology Association. https://www.aad.org/public/diseases/acne/causes/makeup.

